# Monitoring the potential dissemination of antimicrobial resistance in foods, environment, and clinical samples: a one health prospective

**DOI:** 10.1007/s10068-024-01676-z

**Published:** 2024-08-08

**Authors:** Madhuchhanda Das, Anup Kumar Ojha, Karma G Dolma, Tapan Majumdar, Pallab Sarmah, Suranjana Hazarika, Dilem Modi, Dimpu Gogoi, Samaresh Das, Thandavarayan Ramamurthy

**Affiliations:** 1 ICMR, New Delhi, India; 2 Department of Microbiology, Sikkim Manipal Institute of Medical Sciences, Sikkim Manipal University, Gangtok, Sikkim India; 3 AGMC, Agartala, Tripura India; 4 ICMR-RMRC, Dibrugarh, Assam India; 5 GMCH, Guwahati, Assam India; 6 BPGH, Pasighat, Arunachal Pradesh India; 7 C-DAC, Kolkata, India; 8 ICMR-NIRBI, Kolkata, India

**Keywords:** Antimicrobial resistance, Enteric pathogens, Multidrug resistance, One health, Surveillance

## Abstract

Circulation of antimicrobial resistant (AMR) bacteria in the environment, animals, and humans is a major concern. Food chain is an important link to spread AMR across the biosphere. Global warming, preserved and fast foods availability, random use of un-prescribed antimicrobials, unplanned bio-waste management, and using high doses of antibiotics and bio-fertilizers to agricultural fields and animal farms, increase the threat of spreading AMR bacteria. Current study reports prevalence of AMR in enteric pathogens isolated from food, food handlers, environment, and diarrheal patients. The incidence data on AMR pathogens were collected from Assam, Sikkim, Tripura, and Arunachal Pradesh, India, through a well-structured market and hospital surveillance. There  were 428 (3.0%) enteric pathogens detected in food and 184 (5.3%) in clinical samples. Most of the pathogens were resistant (70–100%) to commonly used antimicrobials in treatment of diarrheal infection. Result showed a spread of AMR across food chain, underscoring need for swift preventive intervention.

**Supplementary Information:**

The online version contains supplementary material available at 10.1007/s10068-024-01676-z.

## Introduction

Contaminated foods represent an important vehicle of many infections in humans and animals. In recent times, antimicrobial resistance (AMR) has been a rising concern globally. Environment, plant/animal origin foods, and water play a key role in the spread of AMR bacteria (AMRB) to humans (Samtiya et al., 2022). AMRB can proliferate due to changes in bacterial growth rates and the spread of infectious diseases contributed by global warming (Lio et al., 2023). Furthermore, the AMRB may be selected as a result of the extensive use of preservatives and industrial procedures in the preparation of fast food (Sagar et al., 2023; Samtiya et al., 2022). As the animal gut contains many pathogens, their transmission through animal-origin food cannot be ruled out. Therefore, the food chain is an important link to the transfer of AMRB from environment to human (Founou et al., 2016; Ojha et al., 2023; Thapa et al., 2020; Zarzecka et al., 2020). It is challenging to establish a direct transmission link of the AMRB from the environment or plant/animal-based diets for humans. Hence, the association of inappropriate use of antimicrobials in humans, farm animals, and agricultural fields has been focused in detail (Dutta and Ramamurthy, 2020; György et al., 2021). The One Health concept acknowledges the interdependence of environmental, animal, and human health and the intricate links between each domain. This paradigm is crucial for the extensive use of antibiotics in human healthcare, animal care, and agriculture, which promotes the emergence and spread of AMRB (Asfaw et al., 2022; Fujita et al., 2022; Kahn, 2017; Velazquez-Meza et al., 2022). In view of the complexity to understand AMR transmission, tracing food chains through one health approach of human–animal–environment interface with multidisciplinary action may help to comprehend the AMR transmission (Velazquez-Meza et al., 2022).

A meta-analysis from Ethiopia with pooled AMR prevalence showed the presence of AMRB in 20% of food-producing animals, and 13% in plant-based foods and 69% in milk, and 29% in food handlers and environmental samples. Multidrug resistance (MDR) was very common (74%) among these samples. Common enteric pathogens such as *Staphylococcus* spp. (96%), *Salmonella* spp. (81%), and *Escherichia coli* (77%) showed MDR patterns against commonly used antibiotics in humans and animals (Gemeda et al., 2021). Therefore, prevalence of AMR in common enteric bacteria indicates that enteric bacterial strains are resistant to widely used antibiotics.

In order to establish the possible role of different foods in the spread of AMRB to human, the Indian Council of Medical Research (ICMR) has conducted a multicentric foodborne pathogen surveillance study (ICMR-FoodNet) in Northeast India and collected data of foodborne pathogens and their AMR patterns (Das et al., 2024). In this study, different food samples were collected through market surveillance, clinical samples from diarrheal patients through hospital surveillance and also samples from foodborne outbreak investigations, along with the food handlers’ and environmental samples. As all sets of data are collected from the same area, this comprehensive data may represent the circulation and transmission of AMR pathogens through the food chain. This is one of the larger studies from India, describing the prevalence of enteric pathogens and their AMR patterns from various sources.

## Materials and methods

### Study sites

ICMR conducted a sentinel surveillance in four states of Northeast India, i.e., Sikkim, Assam (Dibrugarh and Guwahati), Arunachal Pradesh (Pasighat), and Tripura (Agartala). Four districts were selected to cover the whole geographical area of each state. One local market and one hospital were designated for each district for the sample collection. Both urban and rural districts were included to cover the diverse food practices of different ethnic groups. As most of the districts are very small, it was presumed that a single supplier may supply food items to all the markets in a district, thus sampling from one main market would represent the entire district. ICMR-FoodNet collaborated with Integrated Disease Surveillance Programme (IDSP) and State Health Officials (SHOs) for this study.

### Sample collection

Several food items, including vegetarian (plant-based), non-vegetarian (animal-based meat/milk), refrigerated, fermented, state-specific ethnic foods, dairy products were selected to maintain uniformity across the centers. Food samples were also collected from restaurants, street food vendors, and shops through market surveillance. Total number of food and water samples tested are shown in Table 1. Suspected food items were selected as per the guidance of the state Food Safety Officer (FSO).

**Table 1:** Number of samples collected and tested by the centers

Sample type	Centre wise samples (n)					
	Dibrugarh, Assam	Guwahati, Assam	Pasighat, Arunachal Pradesh	Agartala, Tripura	Sikkim	Total
Food (vegetarian, non-vegetarian, refrigerated, processed/preserved, and state-specific)	2611	1641	1234	2509	4598	12,593
Clinical samples (stools/rectal swab, and vomitus)	387	118	238	497	2209	3449
Environmental and food handlers	46	19	872	481	169	1587
Total	3044	1778	2344	3487	6976	17,629

Stool/rectal swabs and vomit samples from diarrheal patients were collected from district hospitals through hospital surveillance. Environmental swab samples were taken from working surfaces used for cutting/chopping food items, and surfaces of equipment contaminated by the contact of food items, containers, and refrigerator surfaces. Water used for food processing, hand washing, cleaning, and washing of utensils was collected. Additionally, samples from the stream/local pond water used by the people for cooking/drinking were also collected. Nasal, rectal, and nail bed swab samples for microbial analysis were taken from food handlers who were engaged in food preparation and/or from the persons with poor health conditions.

### Sample processing

Samples were processed and tested for pathogen identification by using the microscopy, cultural and molecular identification methods as per standard operating procedures (ICMR-FoodNet-SOP, 2022). Internal quality control was monitored by the site coordinating center, ICMR-Regional Medical Research Centre, NE (RMRC) Dibrugarh and external quality control was made by the ICMR-National Institute for Research in Bacterial Infections (NIRBI), Kolkata. The identified pathogens were tested for AMR.

Food and waterborne outbreaks were investigated systematically with IDSP and SHOs. Samples (environmental, clinical, food samples) were collected from the outbreak affected areas. All the identified pathogens were phenotypically characterized. Based on the method of disc diffusion (CLSI – Clinical & Laboratory Standards Institute, 2023), a panel of antibiotics used commonly during enteric infections, was tested for each isolated bacterial strain from different sets of samples. The antibiotic disc tested were—ampicillin (AMP; 10 µg/ml), azithromycin (AZI, 15 µg/ml), cefepime (CPM, 30 µg/ml), cefotaxime (CTX, 30 µg/ml), cefoxitin (CXT, 30 µg/ml), ceftazidime (CTZ, 30 µg/ml), ceftriaxone (CTR, 30 µg/ml), cefuroxime (CRX, 30 µg/ml), chloramphenicol (CMP, 30 µg/ml), ciprofloxacin (CIP, 5 µg/ml), clindamycin (CLI, 2 µg/ml), erythromycin (ERY, 15 µg/ml), gentamicin (GEN, 10 µg/ml), imipenem (IMI, 10 µg/ml), linezolid (LNZ, 10 µg/ml), meropenem (MEM, 10 µg/ml), nalidixic acid (NAL, 30 µg/ml), penicillin (PEN, 10 µg/ml), rifampicin (RIF, 5 µg/ml), streptomycin (STR, 10 µg/ml), tetracycline (TET, 30 µg/ml), trimethoprim-sulfamethoxazole (TMP-SMX, 1.25/23.75 µg/ml), and vancomycin (VAN, 30 µg/ml). Briefly, the isolated colonies of pathogenic organisms were selected from primary agar plates in the disc diffusion testing process. To reach 0.5 McFarland turbidity, the inoculum was prepared using either a broth culture or direct colony suspension. Subsequently, Mueller–Hinton agar plates were inoculated, and the antimicrobial discs were placed over the test medium. After incubation, the zone of inhibition size was measured and interpreted using CLSI guidelines (CLSI, Clinical & Laboratory Standards Institute, 2023), to identify the resistance/susceptibility traits.

### Data management/analysis

Data was collected through a digital Case Report Form (CRF). All samples were processed as per the SOP (ICMR-FoodNet-SOP, 2022). Centralized digital data management was organized by the Centre for Development of Advanced Computing (C-DAC), Kolkata. Data analysis was done by using MS Office Excel 365 and Origin Pro 2024 software for spreadsheet organization, statistical calculations, and advanced graphical visualization.

## Results and discussion

From all the five centers, 12,593 food samples, 1587 environmental samples and 3449 clinical samples were collected in this study (Table 1). Details of food, environmental and clinical samples are provided in Table 2. Vegetarian (plant-based), non-vegetarian (animal-based meat/milk), processed or preserved, and state-specific foods, water and environmental and food handlers’ sample were tested for the presence of enteric pathogens from market surveillance study. Environmental samples like water, food station/working surface swabs, cutter scraps, samples like nail bed scrap, and skin swabs from food handlers were also included in the study (Table 2). Similarly, stool, rectal swabs, and vomit samples were tested from hospital surveillance study (Table 2).

**Table 2:** List of clinical, food and environmental samples tested for identification of enteric pathogens

Sample	Type of sample	No. of samples tested	Pathogen identified
Boiled milk	Milk products and sweets (processed)	26	*L. monocytogenes*
Butter		7	ND^a^
Cake		27	*Salmonella* and *S. aureus*
Cheese		69	EPEC, ETEC, and *S. aureus*
Curd		128	*B. cereus,* EPEC, *S. aureus,* and *Y. enterocolitica*
Ghee		8	*S. aureus*
Paneer		42	*V. parahaemolyticus*
Sweet		514	*B. cereus,* EAEC, EPEC, *L. monocytogenes, Salmonella,* and *S. aureus*
Beef	Non-veg (cooked)	155	EAEC, EPEC, *L. monocytogenes, Salmonella, Shigella,* and *S. aureus*
Buffalo		10	*S. aureus*
Chicken		508	*B. cereus,* EAEC, EPEC, *Shigella,* and *S. aureus*
Egg		110	*B. cereus,* and *S. aureus*
Fish		66	*B. cereus,* and *S. aureus*
Mutton		30	EAEC, *Salmonella,* and *S. aureus*
Others		9	ND^a^
Pork		170	*B. cereus,* EAEC, *L. monocytogenes, Salmonella, S. aureus,* and *V. cholerae*
Cold beverages	Refrigerated	1	ND^a^
Ice cream		117	*Shigella*
Milkshake		31	*B. cereus*
Bread	Rice, flour, pulses	29	*S. aureus*
Chhola		73	*S. aureus*
Chowmin		263	*B. cereus,* EAEC, EPEC, and *S. aureus*
Daal		136	*B. cereus,* and *S. aureus*
Dosa /idli		14	*S. aureus*
Momo		246	*B. cereus,* EPEC, and *S. aureus*
Others		21	ND^a^
Puri/paratha/roti		177	*B. cereus,* EAEC, and *S. aureus*
Rice		484	*B. cereus*, *L. monocytogenes,* and *S. aureus*
Chutney	Vegetables	130	*B. cereus,* and EPEC
Cooked vegetables		693	*B. cereus,* EPEC, and *S. aureus*
Fried vegetables/pakoras		262	*B. cereus,* EPEC, and *S. aureus*
Besan batter	Dough and batter	1	ND^a^
Besandough		12	ND^a^
Dosa/idli batter		5	*S. aureus*
Maida dough		285	*B. cereus,* EAEC, EPEC, and *S. aureus*
Rice dough		6	ND^a^
Bamboo shoot	Fermented/processed/preserved	32	ND^a^
Pickles		360	*B. cereus,* EPEC, *Salmonella,* and *S. aureus*
Sauce		11	ND^a^
Soya bean		12	ND^a^
Fruits and vegetables	Fruits, vegetables and salads	2086	*B. cereus,* EAEC, and EPEC, *Salmonella,* and *S. aureus*
Raw milk	Milk products and sweets	296	*B. cereus, Salmonella,* and *Y. enterocolitica*
Beef	Raw/dried meat	160	*B. cereus,* EAEC, EPEC, *Salmonella,* and *S. aureus*
Buffalo		39	EAEC, *Salmonella,* and *S. aureus*
Chicken		1251	*B. cereus,* EAEC, EPEC, ETEC, *L. monocytogenes, Salmonella, Shigella*, *S. aureus*, and *V. parahaemolyticus*
Mutton		232	*Salmonella*, *Shigella* and *S. aureus*
Pork		581	EAEC, EPEC, *Salmonella*, *Shigella*, *S. aureus*, *V. cholerae*, and *V. parahaemolyticus*
Dry fish	Raw/dry fish	389	*B. cereus*, *Salmonella*, *Shigella*, and *S. aureus*
Prawn		1	ND^a^
Raw fish		1595	*B. cereus*, *L. monocytogenes*, *Salmonella*, *Shigella*, *S. aureus*, *V. cholerae*, and *V. parahaemolyticus*
Raw spices	Spices	26	ND^a^
Others	Water	64	*Salmonella* and *V. parahaemolyticus*
Panipuri water		88	*B. cereus* and *S. aureus*
River /pond/stream water		52	*V. parahaemolyticus*
Stored water used for cleaning/washing		85	ND^a^
Tap water		234	*B. cereus*, *Salmonella*, *V. cholerae*, and *V. parahaemolyticus*
Kinema (Sikkim)	State specific	134	*Salmonella* and *S. aureus*
Cooking oil	Environmental	13	ND^a^
Food handler nail bed swab		49	ND^a^
Kitchen clothes		2	ND^a^
Others (cutters scrap)		9	EPEC
Skin swab		218	*S. aureus*
Surface swabs		731	*B. cereus*, EAEC, EPEC, *Salmonella*, *S. aureus*, *V. cholerae*, and *V. parahaemolyticus*
Utensils		257	ND^a^
Water		308	*Shigella* and *V. parahaemolyticus*
Stools, rectal swab, and vomitus	Clinical samples	3449	EAEC, EHEC, EIEC, EPEC, *Salmonella*, *Shigella*, *V. parahaemolyticus*
Total		17,629	

^a^Not detected

### Enteric pathogens identified from food items, environmental and food handlers’ samples

A total of 410 enteric pathogens were isolated from the samples collected from markets. Commonly isolated pathogens were *S. aureus* (n = 152), *B. cereus* (n = 73), diarrheagenic *E. coli* (DEC) such as enteropathogenic *E. coli* (EPEC) (n = 51), enteroaggregative *E. coli* (EAEC) (n = 21), *Salmonella* (n = 59) and *Shigella* (n = 12) (Table S1). From the environmental and food handler’s samples, 18 enteric pathogens were isolated (Table S2).

### Identification of enteric pathogens from clinical samples

A total of 184 enteric pathogens were isolated from diarrheal cases through hospital surveillance. Commonly isolated pathogens were EPEC (n = 103), *Shigella* (n = 31), *Salmonella* (n = 21), and EAEC (n = 20) (Table S3).

### Antimicrobial susceptibility pattern of enteric pathogens identified from market surveillance

Pathogens identified from food samples were tested for antibiotic resistance. Commonly identified bacteria and their AMR profile are depicted in (Table 3 and Fig. S1A). High estimates of resistance were observed in EAEC for ampicillin (62.5%), azithromycin (75%), and cefoxitin (87.5%). In EPEC, high resistance was observed for azithromycin (72.2%) and cefoxitin (72.2%). In ETEC, 50% of resistance was observed for ampicillin, azithromycin, cefotaxime, cefoxitin, ceftazidime, ceftriaxone, ciprofloxacin, imipenem, and nalidixic acid. *Shigella* spp. was mostly resistant to ciprofloxacin (87.5%), ceftriaxone (85.7%) and cefotaxime (71.4%), whereas *Salmonella* showed high resistance to cefotaxime (58.3%). *S. aureus* was susceptible to most of the antibiotics tested.

**Table 3:** Percentage of antibiotic resistance among the enteric pathogens isolated from different food, environmental and food handlers’ samples

Antibiotics	*B. cereus* n^a^ (%)	EAEC n^a^ (%)	EPEC n^a^ (%)	ETEC n^a^ (%)	*Salmonella* n^a^ (%)	*Shigella* n^a^ (%)	*S. aureus* n^a^ (%)
AMP	62 (8.06)	8 (62.5)	18 (38.89)	2 (50)	27 (0)	10 (10)	22 (4.55)
AZI	22 (4.55)	8 (75)	18 (72.22)	2 (50)	29 (13.79)	10 (10)	146 (2.05)
CPM	24 (4.17)	8 (12.5)	18 (5.56)	2 (0)	16 (25)	10 (50)	28 (3.57)
CTX	28 (7.14)	8 (12.5)	18 (16.67)	2 (50)	26 (23.08)	7 (71.43)	26 (3.85)
CXT	26 (11.54)	8 (87.5)	18 (72.22)	2 (50)	12 (58.33)	6 (50)	138 (1.45)
CTZ	24 (8.33)	8 (12.5)	18 (11.11)	2 (50)	27 (25.93)	9 (55.56)	29 (0)
CTR	24 (12.5)	8 (12.5)	18 (11.11)	2 (50)	16 (18.75)	7 (85.71)	21 (14.29)
CRX	21 (14.29)	0 (0)	0 (0)	0 (0)	7 (0)	0 (0)	18 (5.56)
CMP	62 (8.06)	8 (0)	18 (22.22)	2 (0)	14 (7.14)	7 (28.57)	142 (2.82)
CIP	62 (16.13)	8 (25)	18 (33.33)	2 (50)	17 (35.29)	8 (87.5)	147 (6.12)
CLI	57 (3.51)	0 (0)	0 (0)	0 (0)	5 (0)	0 (0)	141 (9.22)
ERY	59 (10.17)	0 (0)	0 (0)	0 (0)	6 (0)	0 (0)	140 (5)
GEN	61 (4.92)	8 (0)	18 (11.11)	2 (0)	34 (8.82)	10 (10)	148 (0.68)
IMI	58 (8.62)	8 (25)	18 (22.22)	2 (50)	28 (10.71)	10 (10)	19 (10.53)
LNZ	21 (28.57)	0 (0)	0 (0)	0 (0)	5 (0)	0 (0)	19 (5.26)
MEM	28 (17.86)	8 (0)	18 (0)	2 (0)	31 (0)	9 (33.33)	18 (11.11)
NAL	21 (4.76)	8 (25)	18 (22.22)	2 (50)	12 (50)	6 (66.67)	20 (10)
PEN	55 (7.27)	0 (0)	0 (0)	0 (0)	6 (0)	0 (0)	21 (9.52)
RIF	51 (9.8)	0 (0)	0 (0)	0 (0)	5 (0)	0 (0)	140 (5.71)
STR	24 (20.83)	0 (0)	0 (0)	0 (0)	6 (0)	0 (0)	21 (14.29)
TET	24 (0)	8 (0)	18 (27.78)	2 (0)	27 (18.52)	8 (0)	147 (0)
TMP-SMX	24 (25)	0 (0)	0 (0)	0 (0)	8 (0)	0 (0)	20 (0)
VAN	59 (10.17)	0 (0)	0 (0)	0 (0)	6 (0)	0 (0)	28 (10.71)

^a^Number of isolates tested

*AMP* ampicillin, *AZI* azithromycin, *CPM* cefepime, *CTX* cefotaxime, *CXT* cefoxitin, *CTZ* ceftazidime, *CTR* ceftriaxone, *CRX* cefuroxime, *CMP* chloramphenicol, *CIP* ciprofloxacin, *CLI* clindamycin, *ERY* erythromycin, *GEN* gentamicin, *IMI* imipenem, *LNZ* linezolid, MEM meropenem, *NAL* nalidixic acid, *PEN* penicillin, *RIF* rifampicin, *STR* streptomycin, *TET* tetracycline, *TMP-SMX* trimethoprim–sulfamethoxazole, *VAN* vancomycin

### Antimicrobial susceptibility pattern of enteric pathogens identified from diarrheal cases

Antibiotic resistance was tested with representative isolates (Table 4). EAEC has shown to have a higher percentage of resistance to cefoxitin (75%), ceftazidime (66.7%), and cefotaxime (64.7%). One EHEC exhibited resistance to azithromycin, cefoxitin, chloramphenicol and gentamicin. All the three EIEC showed resistance to ampicillin, azithromycin, cefepime, cefotaxime, cefoxitin, ceftazidime, ciprofloxacin and nalidixic acid. Isolates of EPEC showed maximal resistance to many of the tested antibiotics (Table 4), while ETEC was resistant (> 75%) to azithromycin, cefoxitin, ceftazidime, ciprofloxacin, gentamicin, and tetracycline. Cefotaxime, ciprofloxacin and nalidixic acid resistance was predominantly seen in *Salmonella* spp., whereas *Shigella* spp. has shown higher resistance to ciprofloxacin.

**Table 4:** Percentage of antibiotic resistance among enteric pathogens isolated from diarrhoeal cases

Antibiotics	EAEC n^a^ (%)	EHEC n^a^ (%)	EIEC n^a^ (%)	EPEC n^a^ (%)	ETEC n^a^ (%)	*Salmonella* n^a^ (%)	*Shigella* n^a^ (%)
AMP	17 (52.94)	1 (0)	3 (100)	46 (100)	4 (50)	18 (27.78)	21 (57.14)
AZI	16 (62.5)	1 (100)	3 (100)	68 (100)	4 (100)	16 (18.75)	18 (22.22)
CPM	17 (58.82)	1 (0)	3 (100)	46 (100)	4 (50)	16 (18.75)	21 (42.86)
CTX	17 (64.71)	1 (0)	3 (100)	49 (100)	4 (50)	17 (35.29)	17 (41.18)
CXT	16 (75)	1 (100)	3 (100)	41 (100)	4 (75)	13 (23.08)	13 (0)
CTZ	15 (66.67)	1 (0)	3 (100)	50 (100)	4 (75)	13 (23.08)	18 (11.11)
CTR	17 (35.29)	1 (0)	3 (66.67)	49 (100)	4 (25)	19 (26.32)	21 (42.86)
CRX	2 (0)	0 (0)	0 (0)	0 (0)	0 (0)	0 (0)	0 (0)
CMP	16 (18.75)	1 (100)	3 (33.33)	23 (100)	4 (25)	13 (0)	17 (17.65)
CIP	17 (52.94)	1 (0)	3 (100)	37 (100)	4 (75)	18 (33.33)	19 (73.68)
CLI	2 (50)	0 (0)	0 (0)	0 (0)	0 (0)	0 (0)	0 (0)
ERY	2 (0)	0 (0)	0 (0)	0 (0)	0 (0)	0 (0)	0 (0)
GEN	17 (41.18)	1 (100)	3 (33.33)	23 (100)	4 (75)	21 (23.81)	23 (26.09)
IMI	16 (37.5)	1 (0)	1 (0)	96 (19.79)	3 (66.67)	13 (0)	8 (50)
LNZ	1 (0)	0 (0)	0 (0)	0 (0)	0 (0)	0 (0)	0 (0)
MEM	18 (5.56)	1 (0)	1 (0)	96 (9.38)	3 (33.33)	16 (6.25)	14 (7.14)
NAL	16 (50)	1 (0)	2 (100)	96 (58.33)	3 (66.67)	11 (36.26)	10 (40)
PEN	2 (0)	0 (0)	0 (0)	0 (0)	0(0)	0 (0)	0 (0)
RIF	2 (50)	0 (0)	0 (0)	0 (0)	0 (0)	0 (0)	0 (0)
STR	2 (0)	0(0)	2 (0)	4 (50)	1 (0)	3 (0)	8 (50)
TET	17 (58.82)	1 (0)	3 (33.33)	33 (100)	4 (75)	15 (33.33)	20 (40)
TMP-SMX	3 (0)	0 (0)	0 (0)	0 (0)	0 (0)	0 (0)	0 (0)
VAN	2 (0)	0 (0)	0 (0)	0 (0)	0 (0)	0 (0)	0 (0)

^a^Number of isolates tested

*AMP* ampicillin, *AZI* azithromycin, *CPM* cefepime, *CTX* cefotaxime, *CXT* cefoxitin, *CTZ* ceftazidime, *CTR* ceftriaxone, *CRX* cefuroxime, *CMP* chloramphenicol, *CIP* ciprofloxacin, *CLI* clindamycin, *ERY* erythromycin, *GEN* gentamicin, *IMI* imipenem, *LNZ* linezolid, MEM meropenem, *NAL* nalidixic acid, *PEN* penicillin, *RIF* rifampicin, *STR* streptomycin, *TET* tetracycline, *TMP-SMX* trimethoprim–sulfamethoxazole, *VAN* vancomycin

Though the proportion of AMR is not the same in clinical and food/environmental enteric pathogen, the resistance patterns of many of them followed a common trend. This includes azithromycin and cefoxitin resistance in EAEC and EPEC, cefoxitin, cefotaxime and ciprofloxacin resistance in *Salmonella* spp. and cefotaxime, ceftriaxone, ciprofloxacin, and nalidixic acid resistance in *Shigella* spp. Identification matching resistance patterns indicate the possible transmission of AMRB within and/or across diarrheal patients/foods/environmental sources.

### Hierarchical cluster heat map antibiogram for AMR status of the pathogens

**Fig. 1:**
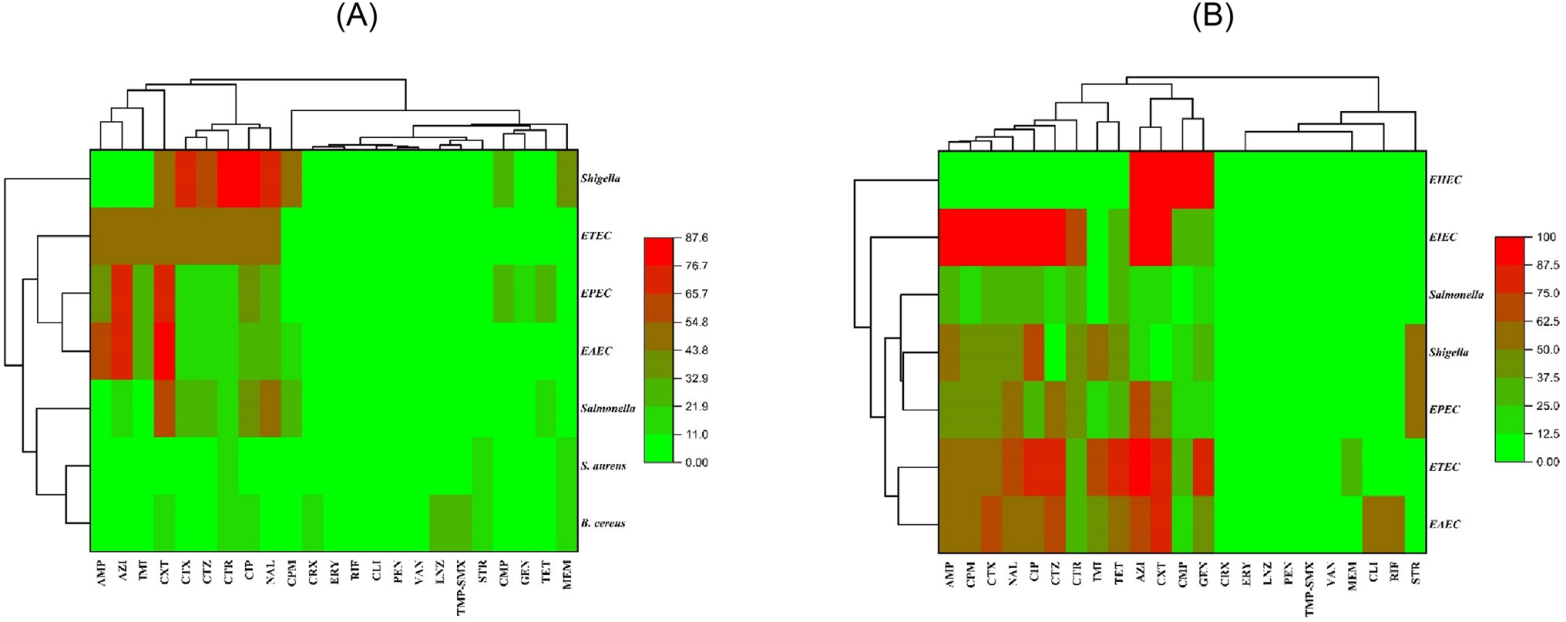
Heatmap antibiogram (**a)** Market surveillance and (**b**) Hospital surveillance. The color from light to dark green shows the percentage (0–50%) of resistance among the isolates while colors from light to dark red show a resistance percentage (50–100%). Rows represent the clusters of pathogens while columns refer to the clusters of antibiotics. *AMP* ampicillin, *AZI* azithromycin, *CPM* cefepime, *CTX* cefotaxime, *CXT* cefoxitin, *CTZ* ceftazidime, *CTR* ceftriaxone, *CRX* cefuroxime, *CMP* chloramphenicol, *CIP* ciprofloxacin, *CLI* clindamycin, *ERY* erythromycin, *GEN* gentamicin, *IMI* imipenem, *LNZ* linezolid, MEM meropenem, *NAL* nalidixic acid, *PEN* penicillin, *RIF* rifampicin, *STR* streptomycin, *TET* tetracycline, *TMP-SMX* trimethoprim–sulfamethoxazole, *VAN* vancomycin

The heatmap antibiograms (hierarchical clustering) of isolates from market and hospital surveillance and their AMR patterns to individual antimicrobials are illustrated in Fig. 1A and B. The clustering dendrograms were constructed by using a hierarchical clustering method and were used in heatmaps to evaluate the AMR determinants (columns) of bacterial isolates (rows). In Fig. 1A, a clear clustering of the DEC (ETEC, EPEC, and EAEC) was observed, where a high resistance pattern was detected for ampicillin, azithromycin, and cefoxitin, which were further aligned in a single cluster in the column. The antibiotics cefotaxime, ceftazidime, ciprofloxacin, and ceftriaxone are aligned in a single cluster showing a pattern of moderate to high resistance for *Shigella*. Similarly, for hospital surveillance of AMR pathogens, high resistance was observed for azithromycin and cefoxitin for DEC arranged in a common cluster of pathogens (Fig. 1B). In EIEC, the cluster of ampicillin, cefepime, cefotaxime, nalidixic acid and ciprofloxacin emerged from a single clade of the dendrogram indicating a higher resistance pattern within this pathotype of DEC. Both hospital and market sample isolates show distinct clusters, but almost similar MDR profiles prevailed in most of the enteric pathogens representing both the groups of samples.

A comprehensive examination of 17,629 distinct samples from a range of human sources and food categories offer valuable insights into the prevalence pathogens and their AMR attributes. Our understanding of the intricate dynamics of AMR transmission is enhanced by large dataset, which was compiled in this study through hospital and market surveillance.

The market surveillance revealed varying AMR profiles of enteric pathogens in different food samples. The large sample size allows for a detailed assessment of resistance patterns across dietary groups, particularly when comparing samples from vegetarian and non-vegetarian food sources. Furthermore, items that are processed, refrigerated, and state-specific food help us better understand how regional variations and storage conditions may affect the AMR trends (Ma et al., 2021; Manyi-Loh et al., 2018; Xu et al., 2022).

The evaluation of patient samples provides an extensive overview of AMR patterns in healthcare settings. The data covering 3,449 clinical samples from the diarrheal cases enables a thorough investigation of AMR patterns of enteric pathogens in human and their possible transmission sources. The clinical implications of this finding highlighted notable incidence of MDR in individuals with diarrheal illnesses. The prevailing AMR indicates how urgently antimicrobial stewardship procedures should be followed in clinical settings (Ahmad et al., 2023; Gajic et al., 2023).

By including environmental samples (water, food handlers’ samples, cutter scrap, and swab from food station/working surface), the study’s purview has been expanded. These samples are essential to understand how food handling practices and environmental factors contribute to the nature and spread of AMR. Food handlers are the important key contributors to the spread of infections. Most notably, the skin swab samples contribute to our understanding of possible reservoirs of AMRB in food handlers. This study shows a possible association of food handlers in the food contamination process. It is crucial to identify AMR pathogens in food handlers to prevent resistant strains from transmitting to the food supply chain (Fujita et al., 2022). In view of the circulation of various resistant pathogens across the food chain, it is important to create awareness related to the spread of AMR among food handlers as well as consumers and stakeholders to protect public health.

Identification of 428 enteric pathogens from food, environmental, and food handlers’ samples indicate the circulation and transmission of a variety of AMRB in the food supply chain. The diversity of enteric pathogens found in these samples is highlighted by the presence of *B. cereus*, DEC (EAEC, EPEC, and ETEC), *Listeria monocytogenes*, *Salmonella* spp., *Shigella* spp., *Staphylococcus aureus*, *Vibrio cholerae*, *V. parahaemolyticus*, and *Yersinia enterocolitica*. Concerns regarding the spread of antibiotic resistance from food to consumers are raised by the notable high incidence of different pathotypes of DEC, which point to possible common sources of contamination. However, *S. aureus* showed a low resistance rate in the AMR testing. Identification of drug resistant pathogens from environmental samples and from food handlers is a warning about the quick spread of AMRB infections.

Based on the hospital surveillance study, 184 enteric bacteria were isolated, which offers important information on the frequency of AMR in the healthcare settings. Considering the difficulties in providing appropriate clinical care and infection control, the existence of AMRB in diarrheal cases is particularly concerning. A possible correlation between gastrointestinal infections and the high frequency of EPEC among hospital isolates highlights the need for targeted interventions in clinical practices. The isolation of *Shigella* spp., and *Salmonella* spp., known to cause severe gastroenteritis with very low infective dose.

In India, diarrheal disease is one of the important causes of death, particularly under 5 years children. In addition, a large number of diarrheal outbreaks are reported every year. High levels of resistance to important drugs, such as ceftazidime, azithromycin etc., was displayed by EAEC, highlighting the difficulties in treating infections driven by this pathotype. One of the EHEC strains was resistant to azithromycin, cefoxitin, chloramphenicol, and gentamicin. Prevalence of EHEC in humans is rare in India.

Evaluation of the antimicrobial susceptibility pattern of pathogens from market surveillance samples offer important information about the effectiveness of various antibiotics against different pathogens linked with common foods. The findings, illustrated in Table 3, and Fig. S1A, highlight the importance of addressing resistance concerns by displaying significant resistance trends among frequently tested microorganisms. The *E. coli* pathotype EAEC exhibited prominent resistance estimates, with ampicillin, azithromycin, and cefoxitin, showing particularly concerning levels of resistance. These results highlight the urgent need for targeted interventions and close observation to address the growing resistance trends. The emergence of AMR against frequently administered antibiotics prompts questions regarding the effectiveness of treatment and highlights the significance of alternative therapeutic approaches. Significant resistance was shown by EPEC, especially to cefoxitin, and azithromycin. Comparably, ETEC showed an alarming resistance to a wide variety of antibiotics, such as imipenem, ciprofloxacin, ampicillin, azithromycin, cefotaxime, cefoxitin, ceftazidime, and nalidixic acid. These results underscore the complex problem of controlling resistance across many pathotypes and the need for customized treatment strategies. The results highlight the importance of ongoing monitoring and the creation of different approaches to treating these clinically important diseases.

Difference in clustering patterns indicates common AMR profiles among pathogens in the hierarchical cluster heat maps (Fig. 1A and B). The unified cluster of DEC (ETEC, EPEC, and EAEC) in Fig. 1A shows strong resistance to ampicillin, azithromycin, and cefoxitin, presenting information on common resistance determinants. Comparable hospital surveillance for DEC with increased resistance to cefoxitin, and azithromycin are depicted in Fig. 1B, emphasizing alarming drug resistance of these enteric pathogens. The dendrogram study highlights the application of hierarchical clustering to find common AMR determinants and direct specific actions (Yudhanto et al., 2022). This approach helps to find clusters of isolates with common AMR determinants in visualizing relationships using tree-like arrangements. By strengthening antimicrobial stewardship, these clusters provide focused surveillance and treatment techniques.

The results underline the critical role of One Health strategy to combat against AMR. Collaborative efforts are necessary to alleviate the multitude of challenges faced by AMR transmission, since, human, animal, and environmental health are interconnected (Asfaw et al., 2022; Fujita et al., 2022; Kahn, 2017; Velazquez-Meza et al., 2022). To track changes in resistance patterns, evaluate the success of interventions, and make necessary strategy adjustments, long-term monitoring is collectively essential. Combining surveillance and appropriate antibiotic use together creates a formidable approach to prevent AMR from spreading further. Healthcare professionals can improve patient outcomes, reduce the emergence of resistant strains, and support global attempts to maintain the effectiveness of antimicrobial drugs by carefully observing patterns of resistance and optimizing antibiotic usage.

**Fig. 2:**
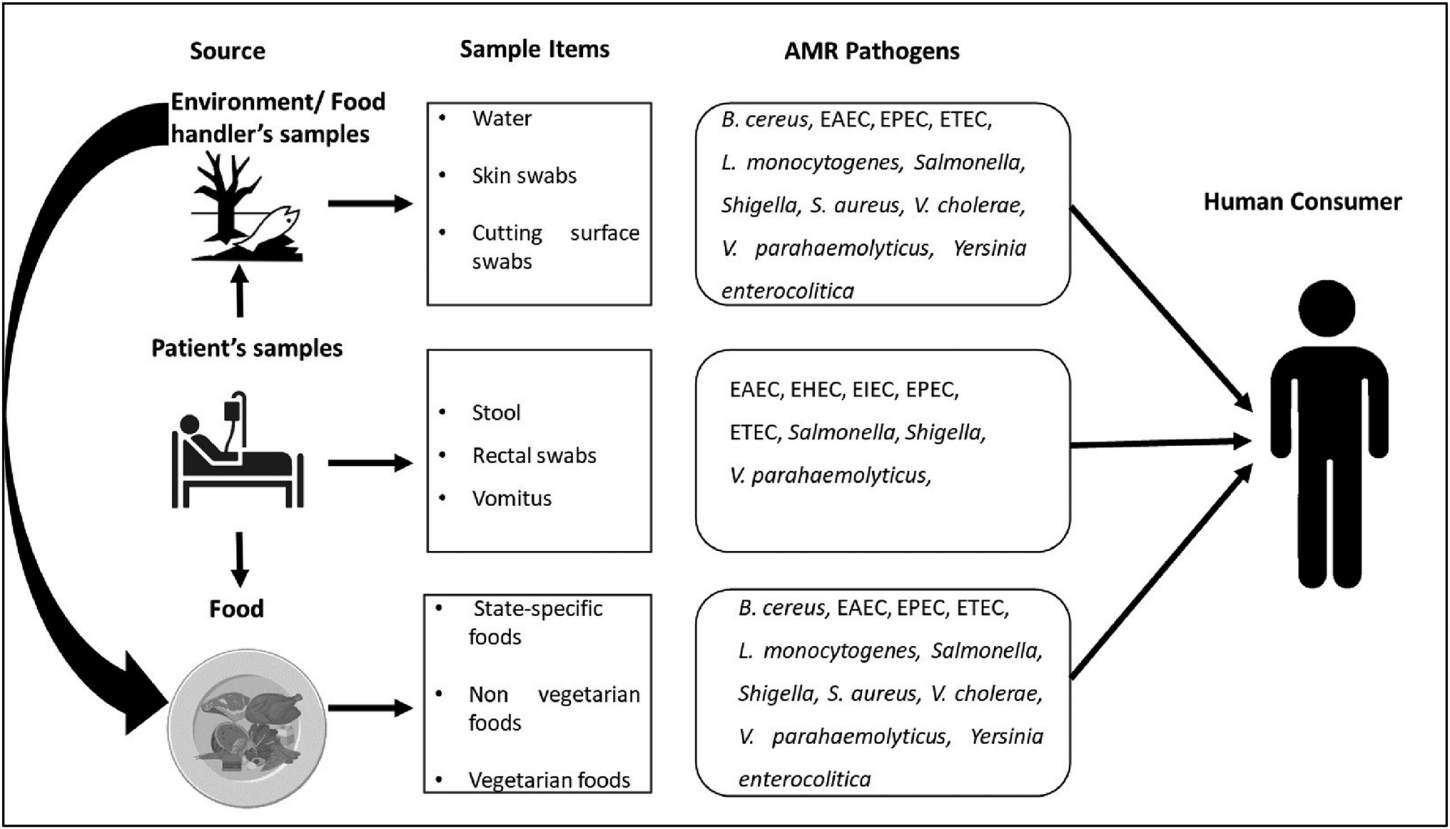
Transmission of AMR through foods to humans

Our findings indicate a current trend in the occurrence of AMR in different food types, environment and diarrheal cases in Northeast India. With this study, we add to the body of information that guides evidence-based interventions aimed at reducing the risk of AMR and preserving public health security. This study also intends to go beyond conventional silos and investigate the interactions between humans, animals, and the environment in the context of AMR transmission by using an integrated One Health approach (Figs. 2, S2). It is important to track unchecked spread of AMRB, as we have limited antibiotics in our hands to treat diseases. The common enteric pathogens like DEC are now showing maximal resistance to all commonly used antibiotics, which is a real concern. Therefore, to protect public health there is a need for One Health approach to control AMR. Although, the study provides very insightful findings to protect human health and makes an alert regarding rising trends of AMR in human–animal–environment interphases. The limitation of this study includes testing of the lower number of pathogenic strains for AMR testing. Molecular tools have not been used to authenticate the transmission of the AMR pathogens. These aspects will be covered in Phase-II study in the other Northeast states of India.

In conclusion, antibacterial drug resistance is a great threat to public health and may lead to major treatment limitations for infectious diseases in the future. Progression in AMR needs urgent actions such as good surveillance and timely interventions, breaking the links of transmission. Appropriate hospital waste management, and judicious use of bio-fertilizer and antibiotics to farm animals are crucial to reduce the AMR spread. The current study showed the spread of AMR across the food chain, underscoring the need for swift preventive intervention. Also, the pathogens identified among food handlers emphasize the need for intense awareness programs and strict monitoring in this pivotal section to enhance food safety and reduce AMR in circulation.

## Supplementary Information

Below is the link to the electronic supplementary material.

Supplementary file1 (DOCX 777 KB)

## Data Availability

All data underlying the results is available as part of the article and no additional source data is required. The data sets used or analyzed during the study will be made available from the corresponding author on reasonable request.
