# Supplementary material for: Monitoring the potential dissemination of antimicrobial resistance in foods, environment, and clinical samples: a one health prospective
Source: Food Sci Biotechnol. 2024 Aug 8;34(3):803–13. doi: 10.1007/s10068-024-01676-z (PMC11822141; doi:10.1007/s10068-024-01676-z)

**Table S1.** Incidence of foodborne pathogens in different food samples

| **Pathogen** | **Sample type** | **No. of Positive sample** | **Incidence%** | **95% CI** |
| --- | --- | --- | --- | --- |
| *B. cereus* | i) Milk products & Sweets (Processed) – curd, and sweet  ii) Non-veg (cooked) – chicken, egg, fish, and pork  iii) Refrigerated – milkshake  iv) Rice, flour, pulses (cooked) – chowmin, daal momo, puri/paratha/roti, and rice  v) Vegetables (cooked/uncooked) – chutney, cooked vegetables, fried vegetables/pakoras  vi) Dough and batter – maida dough  vii)Fermented/processed/preserved – pickles  viii) Fruits, vegetables & salads – fruits, and vegetables  ix) Milk products/sweets (unprocessed) - raw milk  x) Raw/dried meat – beef, chicken, and mutton  xi) Raw/dried fish – raw fish, and dry fish  xii) Water – panipuri water, and tap water | 73 | 0.5148 | (0.5136,0.516) |
| EAEC | i) Milk products & Sweets (Processed) – sweet  ii) Non-veg (cooked) – beef, chicken, mutton, and pork  iii) Rice, flour, pulses (cooked) – chowmin, and puri/paratha/roti  iv) Dough and batter – maida dough  v) Fruits, vegetables & salads – fruits, and vegetables  vi) Raw/dried meat – beef, buffalo, chicken, and pork | 21 | 0.1481 | (0.1475,0.1487) |
| EPEC | i) Milk products & Sweets (Processed) – cheese, curd, and sweet  ii) Non-veg (cooked) – beef, and chicken  iii) Rice, flour, pulses (cooked) – chowmin, and momo  iv) Vegetables (cooked/uncooked) – chutney, cooked vegetables, fried vegetables/pakoras  vi) Dough and batter – maida dough  vii)Fermented/processed/preserved – pickles  viii) Fruits, vegetables & salads – fruits, and vegetables  vi) Raw/dried meat – beef, buffalo, chicken, and pork | 51 | 0.3596 | (0.3587,0.3606) |
| ETEC | i) Milk products & Sweets (Processed) – cheese  vi) Raw/dried meat –chicken | 2 | 0.0141 | (0.0139,0.0143) |
| *L. monocytogenes* | i) Milk products & Sweets (Processed) – boiled milk, and sweet  ii) Non-veg (cooked) – beef, and pork  iii) Rice, flour, pulses (cooked) – rice  iv) Raw/dried meat –chicken  v) Raw/dried fish – raw fish | 11 | 0.0776 | (0.0771,0.078) |
| *Salmonella* | i) Milk products & Sweets (Processed) – cake, and sweet  ii) Non-veg (cooked) – beef, mutton, and pork  iii)Fermented/processed/preserved – pickles  iv) Fruits, vegetables & salads – fruits, and vegetables  v) Milk products/sweets (unprocessed) - raw milk  vi) Raw/dried meat – beef, buffalo, chicken, mutton, and pork  vii) Raw/dried fish – dry fish, and raw fish  viii) Water – others, and tap water  ix) State specific food - kinema | 59 | 0.4160 | (0.415,0.4171) |
| *Shigella* | i) Non-veg (cooked) – beef, and chicken  ii) Refrigerated – ice cream  iii) Raw/dried meat –chicken, mutton, and pork  iv) Raw/dried fish – dry fish, and raw fish | 12 | 0.0846 | (0.0841,0.0851) |
| *S. aureus* | i) Milk products & Sweets (Processed) – cake, cheese, curd, ghee, and sweet  ii) Non-veg (cooked) – beef, buffalo, chicken, egg, fish, mutton, and pork  iii) Rice, flour, pulses (cooked) – bread, chhola, chowmin, daal, dosa/idli, momo, puri/paratha/roti, and rice  iv) Vegetables (cooked/uncooked) –cooked vegetables, and fried vegetables/pakoras  v) Dough and batter – dosa/idli batter, and maida dough  vi)Fermented/processed/preserved – pickles  vii) Fruits, vegetables & salads – fruits, and vegetables  viii) Raw/dried meat – beef, buffalo, chicken, mutton, and pork  ix) Raw/dried fish – raw fish, and dry fish  x) Water – panipuri water  xi) State specific food– kinema | 152 | 1.0719 | (1.0702,1.0736) |
| *V. cholerae* | i) Non-veg (cooked) – pork  ii) Raw/dried meat –pork  iii) Raw/dried fish – dry fish  iv) Water – tap water | 12 | 0.0846 | (0.0841,0.0851) |
| *V. parahaemolyticus* | i) Milk products & Sweets (Processed) – paneer  ii) Raw/dried meat –chicken, and pork  iii) Raw/dried fish – raw fish  iv) Water – others, river/pond/stream water, and tap water | 15 | 0.1058 | (0.1052,0.1063) |
| *Y. enterocolitica* | i) Milk products & Sweets (Processed) – curd  ii) Milk products/sweets (unprocessed) - raw milk | 2 | 0.0141 | (0.0139,0.0143) |
| Total | | 410 |  | |

**Table S2.** Incidence of foodborne pathogens in environmental and food handlers’ samples

| **Pathogen** | **Sample type** | **No. of positive sample** | **Incidence%** | **95% CI** |
| --- | --- | --- | --- | --- |
| *B. cereus* | Surface swabs | 1 | 0.0071 | (0.0069,0.0072) |
| EAEC | Surface swabs | 1 | 0.0071 | (0.0069,0.0072) |
| EPEC | Surface swabs, and cutters scrap | 2 | 0.0141 | (0.0139,0.0143) |
| *Salmonella* | Surface swabs | 1 | 0.0071 | (0.0069,0.0072) |
| *Shigella* | Water | 1 | 0.0071 | (0.0069,0.0072) |
| *S. aureus* | Skin swab, and surface swabs | 3 | 0.0212 | (0.0209,0.0214) |
| *V. cholerae* | Surface swabs | 6 | 0.0423 | (0.042,0.0426) |
| *V. parahaemolyticus* | Surface swabs, and water | 3 | 0.0212 | (0.0209,0.0214) |
| Total | | 18 |  | |

**Table S3.** Detection of enteric pathogens in clinical samples of hospital surveillance

| **Pathogen** | **Sample type** | **Positive sample** | **Incidence**  **%** | **95% CI** |
| --- | --- | --- | --- | --- |
| EAEC | Stool, and swab | 20 | 0.5797 | (0.5772,0.5822) |
| EHEC | Stool | 1 | 0.0290 | (0.0284,0.0296) |
| EIEC | Stool | 3 | 0.0870 | (0.086,0.0879) |
| EPEC | Stool, and swab | 103 | 2.9855 | (2.9798,2.9912) |
| ETEC | Stool | 4 | 0.1159 | (0.1148,0.1171) |
| *Salmonella* | Stool, and swab | 21 | 0.6087 | (0.6061,0.6113) |
| *Shigella* | Stool, and swab | 31 | 0.8986 | (0.8954,0.9017) |
| *V. parahaemolyticus* | Swab | 1 | 0.0290 | (0.0284,0.0296) |
| Total | | 184 |  | |

**Fig S1. Prevalence of AMR pathogens tested for antibiotics from (a) market samples (b) hospital surveillance.**

**a**

**b**

**
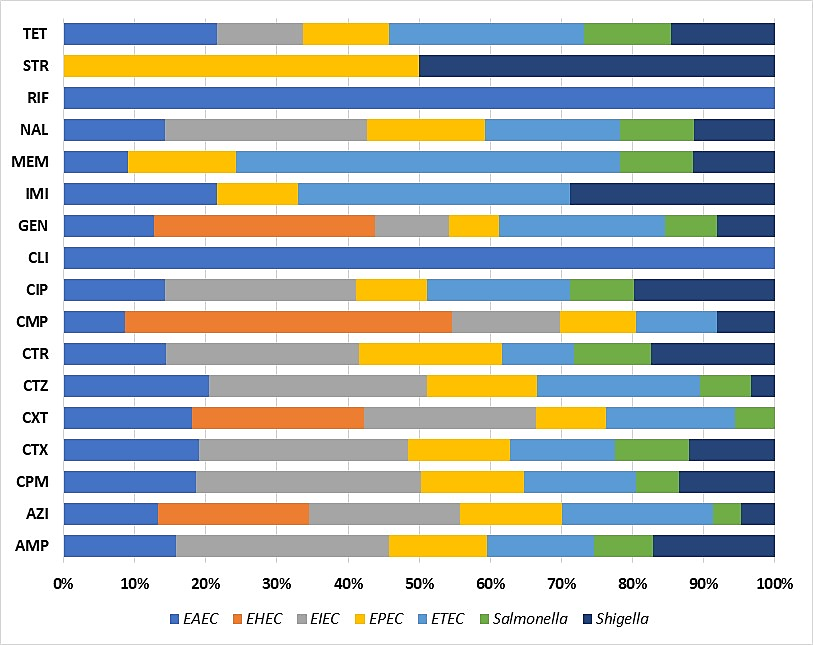

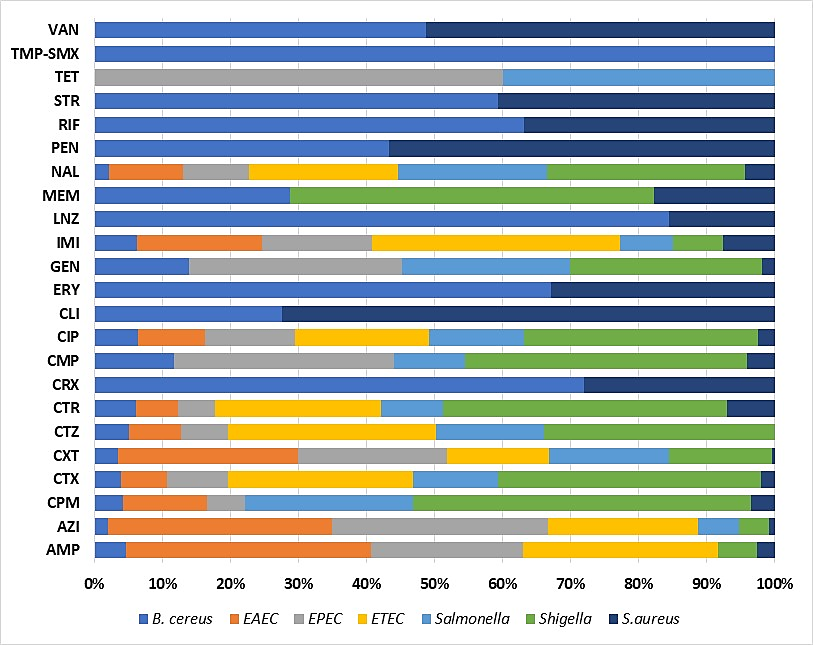
**

**Fig S2. Circulation of AMR pathogens.**


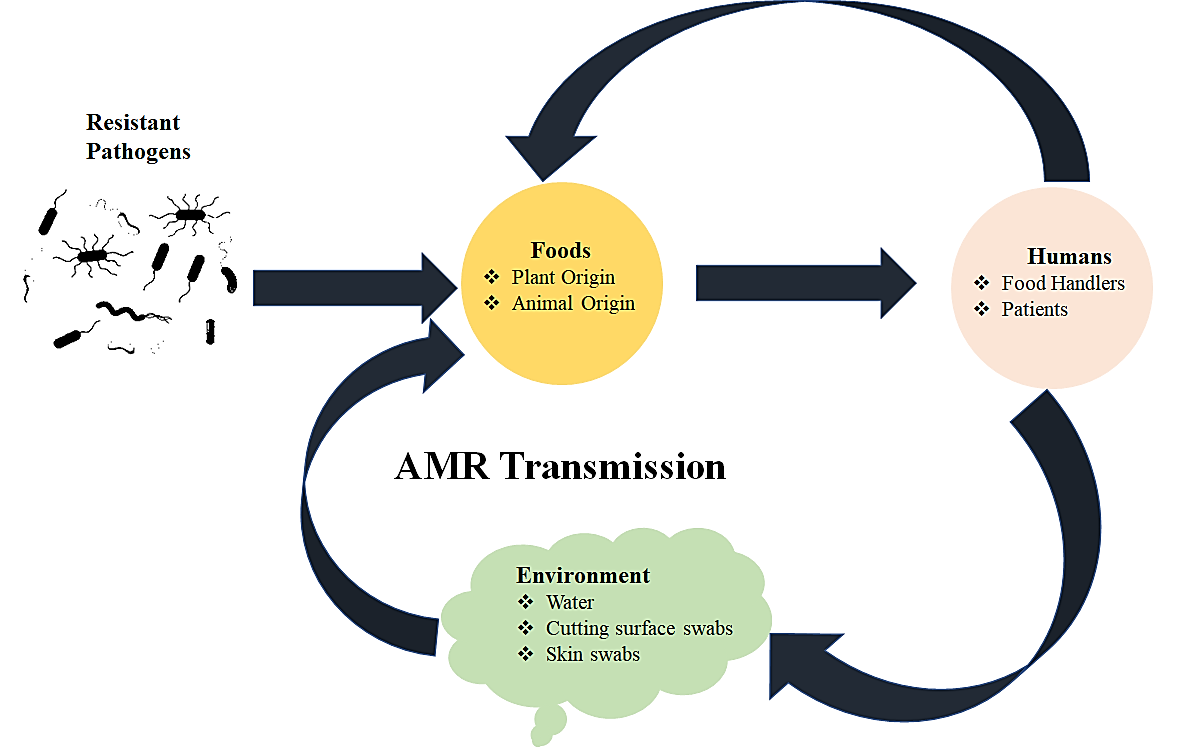

Supplement: Supplementary file 1 — Supplementary file1 (DOCX 777 KB) [file 10068_2024_1676_MOESM1_ESM.docx]
